# An Albumin-Binding PSMA Ligand with Higher Tumor Accumulation for PET Imaging of Prostate Cancer

**DOI:** 10.3390/ph15050513

**Published:** 2022-04-22

**Authors:** Ya’nan Ren, Teli Liu, Chen Liu, Xiaoyi Guo, Feng Wang, Hua Zhu, Zhi Yang

**Affiliations:** 1 Key Laboratory of Carcinogenesis and Translational Research (Ministry of Education/Beijing), Key Laboratory for Research and Evaluation of Radiopharmaceuticals (National Medical Products Administration), Department of Nuclear Medicine, Peking University Cancer Hospital & Institute, Beijing 100142, China; yananren123@126.com (Y.R.); liuteli123321@163.com (T.L.); chanmx@hotmail.com (C.L.); 1911110575@bjmu.edu.cn (X.G.); windtigerwf@163.com (F.W.); 2 School of Medicine, Guizhou University, Guiyang 550025, China

**Keywords:** PSMA, prostate cancer, copper radioisotopes, maleimidopropionic acid, PET imaging

## Abstract

Prostate-specific membrane antigen (PSMA) is an ideal target for the diagnosis and treatment of prostate cancer. Due to the short half-life in blood, small molecules/peptides are rapidly cleared by the circulatory system. Prolonging the half-life of PSMA probes has been considered as an effective strategy to improve the tumor detection. Herein, we reported a ^64^Cu-labeled PSMA tracer conjugating with maleimidopropionic acid (MPA), ^64^Cu-PSMA-CM, which showed an excellent ability to detect PSMA-overexpressing tumors in delayed time. Cell experiments in PSMA-positive 22Rv1 cells, human serum albumin binding affinity, and micro-PET imaging studies in 22Rv1 model were performed to investigate the albumin binding capacity and PSMA specificity. Comparisons with ^64^Cu-PSMA-BCH were performed to explore the influence of MPA on the biological properties. ^64^Cu-PSMA-CM could be quickly prepared within 30 min. The uptake of ^64^Cu-PSMA-CM in 22Rv1 cells increased over time and it could bind to HSA with a high protein binding ratio (67.8 ± 1.5%). When compared to ^64^Cu-PSMA-BCH, ^64^Cu-PSMA-CM demonstrated higher and prolonged accumulation in 22Rv1 tumors, contributing to high tumor-to-organ ratios. These results showed that ^64^Cu-PSMA-CM was PSMA specific with a higher tumor uptake, which demonstrated that MPA is an optional strategy for improving the radioactivity concentration in PSMA-expressing tumors and for developing the ligands for PSMA radioligand therapy.

## 1. Introduction

According to the 2018 global cancer statistics, prostate cancer (PCa) ranks second in male cancer incidence and fifth in mortality [1]. Due to its high expression in prostate cancer cells, prostate-specific membrane antigen (PSMA) has been reported as an effective target for specific imaging and targeted therapy of prostate cancer [2]. In the past 20 years, extensive small-molecule inhibitors of PSMA have emerged, which demonstrated high binding affinity and PSMA specificity. Among them, radiolabeled small molecules/peptides including ^68^Ga/^177^Lu/^225^Ac-PSMA-617, ^68^Ga/^177^Lu/^225^Ac-PSMA-I&T, ^68^Ga-PSMA-11, ^99m^Tc-MIP-1404, ^99m^Tc-PSMA-I&S, ^18^F-DCFPyL, Al^18^F-PSMA-BCH, and ^18^F-PSMA-1007 currently dominate in clinical trials for PCa imaging or PSMA radioligand therapy (PRLT) [3,4,5,6,7,8,9,10,11,12]. In recent years, PRLT has seen a pandemic of metastatic castration-resistant prostate cancer (mCRPC) lesions, leading to at least a 50% reduction in prostate-specific antigens and showing an acceptable safety profile with negligible side effects [13]. However, as a small molecule compound, the fast clearance in blood and the particularly insufficient dose delivery to tumors leads to higher dosage administration or more frequent treatments, which may increase the possibility of adverse reactions and limit [14] the effectiveness of β-particle radiotherapy [14].

In order to improve the utilization efficiency of nuclides and enhance the tumor inhibition efficiency, some strategies for albumin binding have been developed to prolong the blood circulation time and increase radioactive accumulation in tumors [15,16]. Human serum albumin (HSA, MW 66 kDa), the most abundant protein in plasma, composed of seventeen pairs of disulfide bonds and one free cysteine (cysteine-34), is known to be a versatile carrier for drug targeting [17,18]. There has been long-term interest in improving the in vivo pharmacokinetic properties of small-molecule drugs or imaging probes by utilizing albumin’s long physiologic half-life (19 days), thereby enhancing therapeutic effect. Cysteine-34, the only free thiol group in albumin, located in the hydrophobic crevice, holds an excellent chemical characteristic for further structural modification to produce novel bioactive constructs with an extended half-life [19,20,21]. For instance, when conjugated with 4-(P-iodophenyl) butyric acid and Evans blue—both of which can bind to albumin—PSMA inhibitors could availably improve the accumulation in kidneys and increase the uptake in tumors with PSMA positive expression [15,22]. 

Maleimidopropionic acid (MPA) modification provides another attractive platform for targeted drug delivery, allowing for binding with albumin cysteine-34 specifically and covalently to form a thiosuccinimide bond [23]. Several successful combinations have demonstrated the benefits of combining macromolecular therapies. Timms et al. [24] confirmed that the presence of the reactive maleimidopropionic acid group, which covalently linked the peptide CJC-1295 to free cysteine-34 in albumin, could significantly prolong the activity retention of peptides in the bloodstream. Xie et al. [25] chemically modified the peptide of HIV fusion inhibitor with a single MPA exhibiting potent anti-HIV activity and a remarkably extended half-life in vivo. Feng et al. [26] developed an MPA-conjugated peptide which exhibited a prolonged half-life and enhanced anti-tumor efficiency in vivo. 

We recently also reported a novel albumin-based PSMA probe Al^18^F-PSMA-CM. The probe was modified with MPA, which showed higher PSMA specificity and longer half-life in blood, contributing to a distinct accumulation in 22Rv1 tumors, with increasing uptake in 4 h [11,27]. These results indicated MPA may be a promising choice to develop radiopharmaceuticals for PRLT of prostate cancer. But the half-life of ^18^F was not enough to match the circulation of MPA-conjugated PSMA-CM, which cannot evaluate the efficiency of PSMA-CM. Compared with ^18^F (T_1/2_, 109.77 min; β^+^, 96.7%), ^64^Cu (T_1/2_, 12.7 h; β^+^, 17.4% [E_max_, 0.656 MeV]; β^−^, 39% [E_max_, 0.573 MeV]) was considered to be a diagnostic nuclide with its unique decay characteristics of the matched pair ^64^Cu/^67^Cu for theranostic applications. In addition, the longer half-life of ^64^Cu provides an advantage for delay imaging, which may effectively monitor the uptake of radiotracers in tumors [28]. Hence, in order to match the prolonged half-life of PSMA-CM, we radiolabeled PSMA-CM with ^64^Cu and evaluated ^64^Cu-PSMA-CM in mice bearing 22Rv1 tumors, showing great potential for detecting prostate lesions in delayed imaging.

## 2. Results

### 2.1. Radiosynthesis and Quality Control

**Figure 1:**
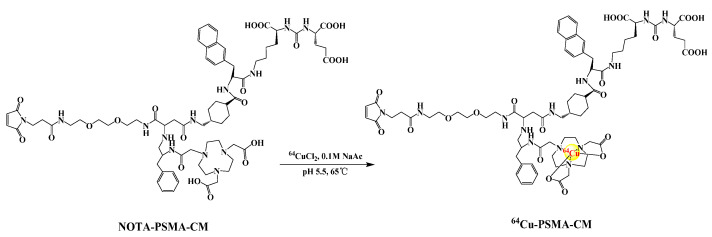
The radio-labeling process of ^64^Cu-PSMA-CM.

The radiolabeling process of ^64^Cu-PSMA-CM is depicted in Figure 1. The radiochemical yield of ^64^Cu-PSMA-CM was over 75% and radiochemical purity was >99% after a Sep-Pak C18 cartridge purification (Figure S1). The specific activity was 15.97 ± 2.01 GBq/μmol.

### 2.2. In Vitro Stability

**Figure 2:**
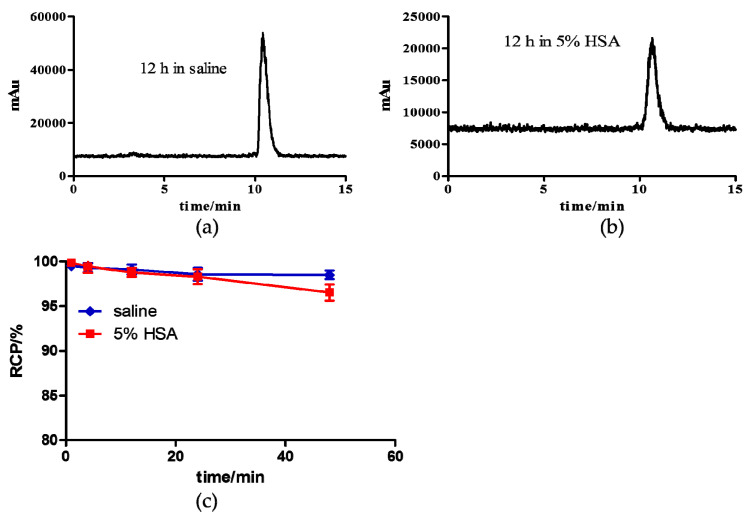
In vitro stability of ^64^Cu-PSMA-CM. The radio-HPLC patterns of (**a**) ^64^Cu-PSMA-CM after incubation in saline and (**b**) 5% HSA for 12 h. (**c**) The radio-chemical purity of ^64^Cu-PSMA-CM in saline (blue) and 5% HSA (red) at 1 h, 4 h, 12 h, 24 h, and 48 h.

The in vitro stability of ^64^Cu-PSMA-CM was tested in 5% human serum albumin (HSA) and saline at 37.0 °C. As shown in Figure 2, the radiochemical purity (RCP) of ^64^Cu-PSMA-CM was over 95% after more than 48 h incubation in saline and 5% HSA, indicating that ^64^Cu-PSMA-CM was quite stable and can be applied for further study.

### 2.3. Partition Coefficient

The partition coefficient of ^64^Cu-PSMA-CM was calculated, and the log *p*-value was −2.25 ± 0.05, indicating that ^64^Cu-PSMA-CM was highly hydrophilic.

### 2.4. Western Blotting and In Vitro Cell Experiments

**Figure 3:**
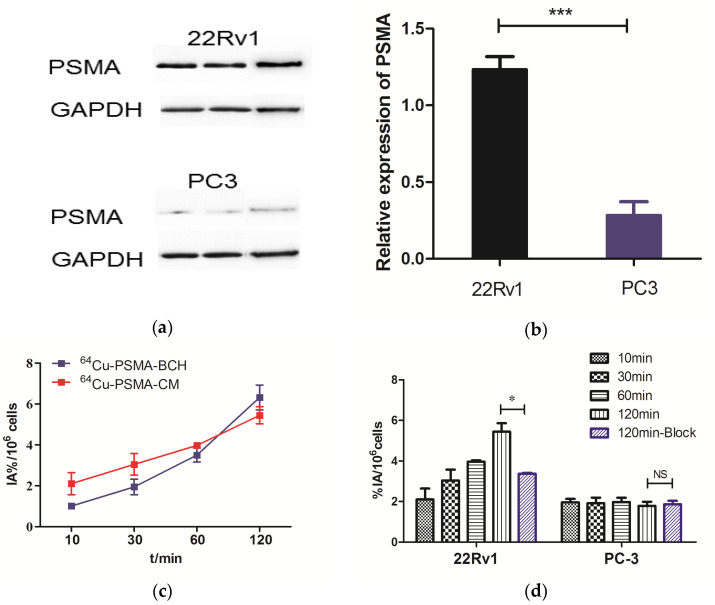
(**a**,**b**) were relative PSMA expression in 22Rv1 and PC3 cell lines. The expression of PSMA in 22Rv1 cells was significantly higher than in PC3 cells. The cell uptake of ^64^Cu-PSMA-BCH and ^64^Cu-PSMA-CM in (**c**) 22Rv1 cells or (**d**) PC-3 cells. *: *p* < 0.05, ***: *p* < 0.001, NS = not statistically significant. Block = 1 μg of ZJ-43.

The expression of PSMA in 22Rv1 and PC3 cells were measured by a Western blot assay. The 22Rv1 cells showed significantly higher expression level of PSMA than PC3 cells with relative values of 1.23 ± 0.07 and 0.283 ± 0.07 (*p* = 0.00018), which indicates the high expression of PSMA in 22Rv1 cells (Figure 3a,b).

Cell uptake experiments were performed on PSMA (+) 22Rv1 and PSMA (−) PC-3 cells, as shown in Figure 3c. The uptake of ^64^Cu-PSMA-CM in 22Rv1 cells increased with time, it was comparable to ^64^Cu-PSMA-BCH (5.45 ± 0.42 %IA/10^6^ cells vs. 6.31 ± 0.60 %IA/10^6^ cells at 120 min, respectively). The uptake in 22Rv1 cells can be significantly blocked by ZJ-43 (blocked to 3.38 ± 0.04 IA%/10^6^ cells, *p* = 0.02). However, the uptake values of ^64^Cu-PSMA-CM in PC-3 cells were lower than that in 22Rv1 cells, and the uptake cannot be blocked by excess of ZJ-43 (*p* > 0.05) (Figure 3d).

### 2.5. Binding Affinity Assay

Human serum albumin binding assay was performed to test the albumin binding ability of ^64^Cu-PSMA-CM. The protein binding ratio of ^64^Cu-PSMA-BCH and ^64^Cu-PSMA-CM were calculated as 55.9 ± 4.0% and 67.8 ± 1.5% (*p* = 0.003).

**Figure 4:**
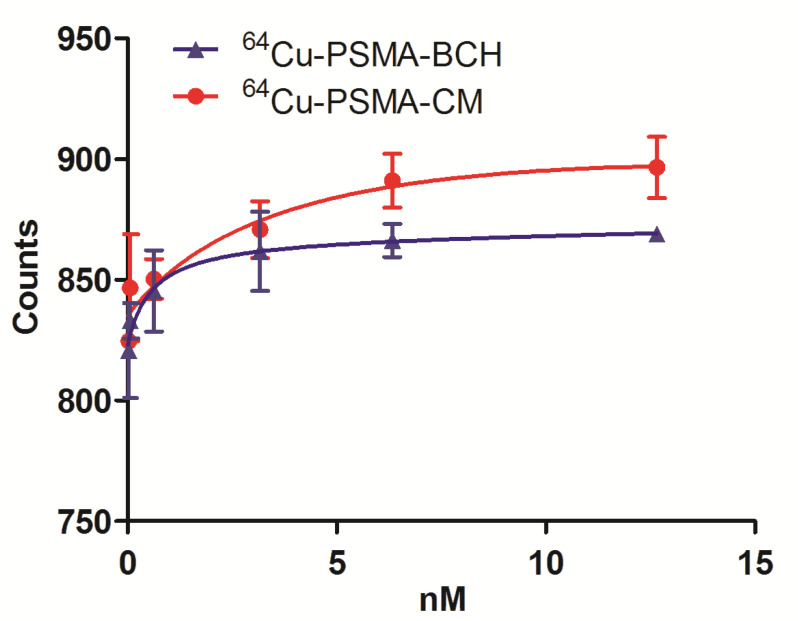
The binding affinity of ^64^Cu-PSMA-CM and ^64^Cu-PSMA-BCH to PSMA in 22Rv1 cells. The dissociation constant values of ^64^Cu-PSMA-BCH and ^64^Cu-PSMA-CM were 0.59 nM and 4.58 nM, respectively.

The binding affinity of ^64^Cu-PSMA-CM and ^64^Cu-PSMA-BCH to PSMA were conducted on 22Rv1 cells. The dissociation constant values of ^64^Cu-PSMA-BCH and ^64^Cu-PSMA-CM were 0.59 nM and 4.58 nM, respectively (Figure 4).

### 2.6. Pharmacokinetics

**Figure 5:**
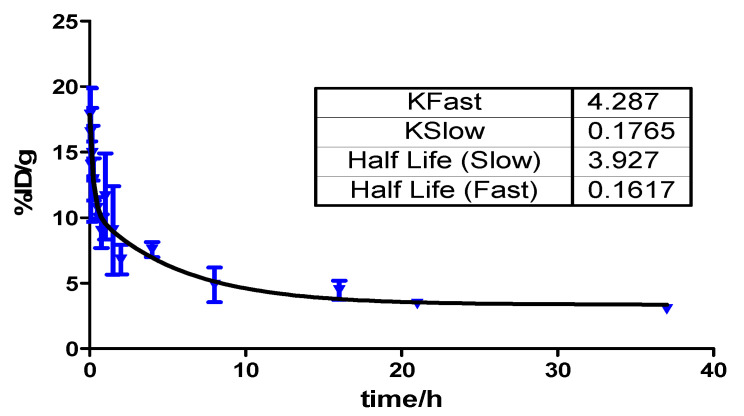
Blood activity–time profile of ^64^Cu-PSMA-CM in normal Kunming mice. The half-life of the distribution phase and elimination phase were 0.16 h and 3.93 h, respectively.

The pharmacokinetics profile of ^64^Cu-PSMA-CM in blood circulation was tested by injecting 3.7 MBq of ^64^Cu-PSMA-CM into Balb/c male mice (n = 4). As shown in Figure 5, the blood activity–time profile of ^64^Cu-PSMA-CM was: *Ct =* 7.21*e*^−4.287t^ *+* 7.29*e*^−0.176t^. The half-life of the distribution phase and elimination phase were 0.16 h and 3.93 h, respectively, which significantly delayed the elimination phase compared to ^64^Cu-PSMA-BCH (0.208 min) [29].

### 2.7. Biodistribution Studies

**Figure 6:**
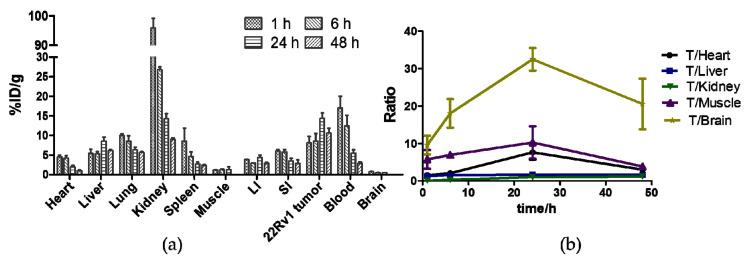
(**a**) Bio-distribution of ^64^Cu-PSMA-CM in 22Rv1 mice at 1 h, 6 h, 24 h, and 48 h. (**b**) Tumor-to-organ ratios according to the distribution. LI—large intestine; SI—small intestine; T—tumor.

The biodistribution studies of ^64^Cu-PSMA-CM were performed in 22Rv1-beared male mice. As shown in Figure 6a and Table S1, within 1 h, ^64^Cu-PSMA-CM rapidly accumulated in the blood pool (16.95 ± 3.05 ID%/g), liver (5.34 ± 1.13 ID%/g), and kidneys (95.84 ± 3.33 %ID/g). Subsequently, the clearance in the kidneys was fast with uptake values of 8.92 ± 0.41 %ID/g at 24 h p.i. and was accompanied by the decline of radioactivity in other organs, such as heart, lung, spleen, and blood, demonstrating that ^64^Cu-PSMA-CM was mainly excreted from the urinary system. The uptake in 22Rv1 tumor was increased within 24 h with a highest uptake value of 14.29 ± 1.44 %ID/g at 24 h p.i., and it decreased at 48 h p.i., advantageously providing a high tissue contrast at 24 h p.i. (Figure 6b). 

### 2.8. Micro-PET Imaging

**Figure 7:**
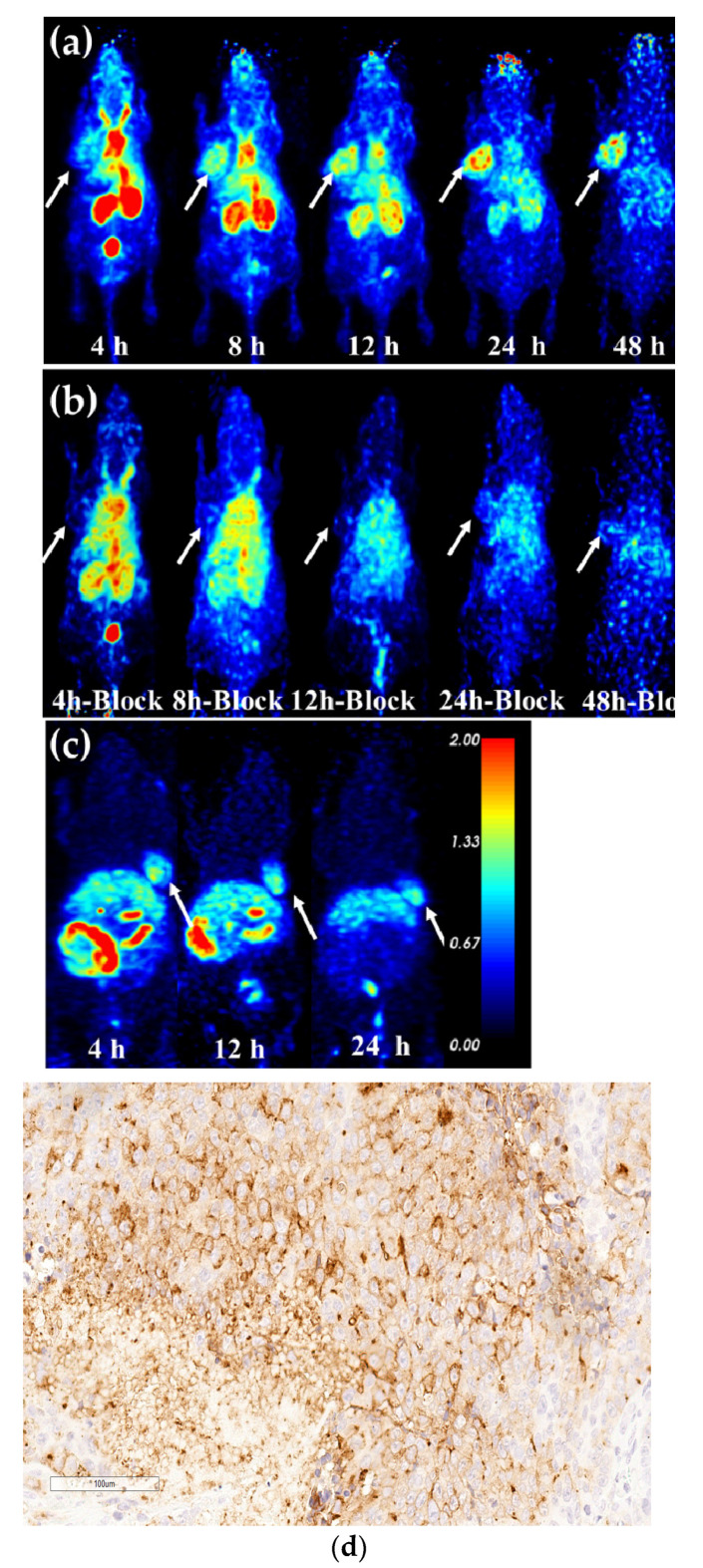
Micro-PET images of ^64^Cu-PSMA-CM and comparison with ^64^Cu-PSMA-BCH in 22Rv1 xenograft nude mice. Maximum intensity projection (MIP) images of ^64^Cu-PSMA-CM in mice bearing 22Rv1 tumors (**a**) without or (**b**) with the co-injection of blocker ZJ-43 at 4 h, 8 h, 12 h, 24 h, and 48 h p.i., and (**c**) images of ^64^Cu-PSMA-BCH at 4 h p.i. 12 h and 24 h p.i. in mice bearing 22Rv1 tumors. White arrows indicated 22Rv1 tumors. Immunohistochemical staining of (**d**) 22Rv1 xenografts tumor showed PSMA-positive (20 × amplifcation).

Micro-PET imaging of ^64^Cu-PSMA-CM and a comparison with ^64^Cu-PSMA-BCH were demonstrated on mice bearing 22Rv1 tumors. As shown in Figure 7a and Figure S2a, the heart, liver, kidneys, salivary glands, and 22Rv1 tumors were observed. Kidneys had the highest uptake of ^64^Cu-PSMA-CM within 12 h, followed by the heart and liver, and the radioactivity of these organs rapidly decreased over time. The accumulation in 22Rv1 tumors continuously increased until 24 h p.i. and then decreased at 48 h, which was similar to the results of previous biodistribution studies. When co-injected with excess ZJ-43 (50 μg), the uptake in tumors and kidneys significantly decreased (*p* < 0.001). Due to the fast clearance of ^64^Cu-PSMA-CM from the nontumor organs, relatively selective tumor images become quite clear at 12 h, and good contrast images in tumors was obtained at 24 h. Generally, the tumor-to-non-target organ ratios of ^64^Cu-PSMA-CM increased with time, and the excellent tumor-to-kidneys ratio (1.29 ± 0.05) and tumor-to-liver ratio (1.76 ± 0.08) were at 24 h p.i. (Figure S3). When compared to ^64^Cu-PSMA-BCH, a tracer without MPA, ^64^Cu-PSMA-CM demonstrated prolonged circulation in vivo and significantly higher uptake in 22Rv1 tumor and organs, such as heart, liver, and kidneys (Figure 7a,c and Figure S2). As previously mentioned, ^64^Cu-PSMA-CM showed the highest accumulation in 22Rv1 tumor with an SUV mean value of 1.88 ± 0.04 at 24 h p.i., whereas the peak uptake of ^64^Cu-PSMA-BCH was 0.84 ± 0.02 at 4 h p.i. (Figure S2). In addition, due to the fast clearance of ^64^Cu-PSMA-BCH from the kidneys, the tumor-to-kidney ratio was higher than ^64^Cu-PSMA-CM, while the tumor-to-liver ratio of ^64^Cu-PSMA-CM was superior to ^64^Cu-PSMA-BCH (Figure S3). Furthermore, immunohistochemical staining results indicated the positive expression of PSMA in 22Rv1 tumor tissue (Figure 7d), further confirming the targeted accumulation of the probe in 22Rv1 tumor.

### 2.9. Estimation of Radiation Dosimetry

Human organ radiation dosimetry was estimated based on the biodistribution data in mice bearing 22Rv1 tumors. As shown in Table 1, the liver received the highest absorbed dose (0.107 mGy/MBq), followed by the kidneys and gallbladder wall with absorbed doses of 0.0997 mGy/MBq and 0.0414 mGy/MBq, respectively. The effective dose of whole body is 0.0276 mSv/MBq, which was similar to ^64^Cu-PSMA-BCH (0.0292 mSv/MBq) [29].

## 3. Discussion

Due to the fast clearance in blood and particularly insufficient dose delivery to tumor of small molecule compounds ^177^Lu/^225^Ac-PSMA-617, there have been long-term efforts in developing a general strategy which can effectively prolong the compounds’ half-life in vivo. Among the reported methods, the combination of pharmaceuticals and albumin-binding molecules is one of the most commonly used methods, which has high efficiency and few side effects [17]. In the past five years, many albumin-based PSMA probes have been developed, but most of the studies focused on 4-(p-iodophenyl)butyric acid and Evans blue derivatives, aiming to improve metabolic behavior in the body [22,30,31,32]. The clinical studies of ^177^Lu-EB-PSMA-617 had been performed and achieved promising results in the therapy of mCRPC [21,32]. We previously confirmed maleimidopropionic acid (MPA) modification, offering an another effective platform for binding albumin and conjugating with the PSMA tracer [27]. Here, benefiting from the moderate half-life of copper-64, we developed a ^64^Cu-labeled MPA-modified PSMA tracer and evaluated the targeting capability of the PSMA-tumor.

^64^Cu-PSMA-CM was prepared with high radiochemical yield, high radiochemical purity, and moderate specific activity. It was quite stable in vitro, which meets the criteria of quality control and can be used for further studies.

When compared with ^64^Cu-PSMA-BCH, a PSMA probe without MPA moiety, ^64^Cu-PSMA-CM showed a higher human serum albumin binding ratio and longer half-life. This indicated that the conjugation of MPA indeed enhanced the binding ability of the tracer to HSA and extended the circulation in blood, which was expected to offset the defect of the rapid clearance of small molecule inhibitors. Although the binding affinity of ^64^Cu-PSMA-CM to PSMA decreased, the Kd value was still at the level of nanomole, which was comparable with other reported PSMA probes.

^64^Cu-PSMA-CM was specifically accumulated in PSMA (+) 22Rv1 cells and increased over time with the highest uptake value of 5.45 ± 0.42 IA%/10^6^ cells at 120 min, which was significantly higher than that in PSMA (−) PC-3 cells (1.79 ± 0.20 IA%/10^6^ cells). It can be specifically blocked to 3.38 ± 0.04 IA%/10^6^ cells by PSMA inhibitor, ZJ-43, indicating the high specificity of ^64^Cu-PSMA-CM to PSMA. The comparable uptake values of ^64^Cu-PSMA-CM and ^64^Cu-PSMA-BCH in PSMA (+) 22Rv1 cells indicated that the conjugation of MPA moiety had little effect on the specificity and binding affinity, or for the uptake in cells.

Both biodistribution and micro-PET imaging studies in 22Rv1-beared mice confirmed that ^64^Cu-PSMA-CM was highly accumulated in PSMA (+) tumors and washed out from normal organs at delayed time points. This resulted in a continuous increase of tumor-to-organ ratio, prospectively facilitating the observation of more tumor lesions. longer circulation in the blood resulted in longer retention of radioactivity in the kidneys and liver. 

When compared with ^64^Cu-PSMA-BCH, 22Rv1 tumors showed excellent permeability and retention of ^64^Cu-PSMA-CM, which may improve the contrast of tumors to non-target organs. The higher uptake in tumors and comparable uptake in cells indicated that the higher uptake in PSMA (+) positive tumors was due to the prolonged circulation of ^64^Cu-PSMA-CM in the blood. In addition, the significantly higher tumor accumulation in delayed time demonstrated that the compound fulfilled the prerequisites for dosimetry in the course of therapy planning with ^67^Cu. It also showed that modification with MPA is a good strategy to improve radioactivity accumulation in PSMA (+) tumors, which may effectively guide the development of ^177^Lu or ^225^Ac labeled tracers for PRLT.

## 4. Materials and Methods

### 4.1. General Materials

^64^Cu was produced by the HM-20 cyclotron via ^64^Ni (p,n) ^64^Cu reaction at Beijing Cancer Hospital. Human serum albumin (HSA) was purchased from Chengdu Rongsheng Pharmaceuticals Co., Ltd. (Chengdu, China). ZJ-43 (N-[[[(1S)1-Carboxy-3-methylbutyl]amino]-carbonyl]-L-glutamic acid) was purchased from Tocris Bioscience (Bristol, UK). The precursor, NOTA-PSMA-BCH (BCH is Beijing Cancer Hospital) and PSMA-CM (CM stands for Covalent binding with Maleimidopropionic acid) were acquired from the China Peptides Company. Acetonitrile and trifluoroacetic acid (TFA) were purchased from Honeywell International Inc. (Elmsford, New York, NY, USA) and Shanghai Aladdin Biochemical Technology Co., Ltd. (Shanghai, China), respectively. Sep-Pak C18-Light cartridges were purchased from Waters (Leinster, Ireland). The Radio-TLC was conducted on an AR 2000 system (Bioscan, Poway, CA, USA). Filter paper was used as carrier and the eluant was a mixture of saturated EDTA and saline. Micro-PET was performed on Super Nova PET/CT (PINGSENG, Shanghai, China).

### 4.2. Radiosynthesis and Quality Control of ^64^Cu-PSMA-CM

The precursor, NOTA-PSMA-CM, was synthesized using a solid phase platform. Briefly speaking, the PSMA-targeting urea-based pharmacophore _L_-Glu-NH-CO-NH-_L_-Lys- was protected by resinimmobilized ε-allyloxycarbonyl lysine. The dehydrated product of 3-maleimidopropionic acid (3-(2,5-dioxo-2,5-dihydro-1H-pyrrol-1-yl)propanoic acid) and 1-hydroxy-1h-pyrrole -2,5-dione reacted with 2,2’-(ethane-1,2-diylbis(oxy))bis(Ethan-1-amine) to form substrates 1-(3-methylene-6-(2-propoxyethoxy) hexyl) 1*H*-pyrrole-2, 5-dione3-Maleimidopropionic acid. And then, the compound was reacted with NOTA-chelated 2,5-dioxo-2,5-dihydro-1*H*-pyrrol-1-yl4-(allyloxy)-2-(2-formamido-3-phenylpropanamido)pent-4-enoate. After the catalytic reaction of tetrakis(Triphenylphosphine)palladium and benzylamine, the compound was mixed with the reagents of Fmoc-2-Nal-OH (2-({[(9H-fluoren9-yl)methoxy]carbonyl}amino) -3-(naphthalen-2-yl), Fmoc-tranexamic acid {(1r,4r)-4-[({[(9H-fluoren-9- yl)methoxy]carbonyl}amino)methyl]cyclohexane-1-carboxylic acid} and then 2,2’- (7- (4-benzyl-7-(2-((((1S, 4s)–4-(((R)-1–carboxy–2-(naphthalen–2-yl)ethyl)carbamoyl) cyclohexyl)methyl)amino)–2-oxoethyl)–21-(2,5–dioxo-2,5-dihydro-1*H*-pyrrol-1-y)-2,8,19-trioxo-12,15-dioxa-3,6,9,18-tetraazahenicosyl)-1,4,7-triazonane-1,4-diyl)diacetic acid was obtained. Finally, the compound was reacted with PSMA-targeting urea-based pharmacophore _L_-Glu-NH-CO-NH-_L_-Lys- by a series of chemical reactions and carboxyl deprotection to obtain the final product, (((1S)–5-((2R)–2-((1s,4S)–4 -(5-((2-(2-(4,7-bis(carboxymethyl)-1,4,7–triazonan–1-yl)acetamido)–3-phenylpropyl)amino)–19-(2,5-dioxo-2,5-dihydro-1H–pyrrol–1-yl)-3,6,17–trioxo-10,13–dioxa-2,7,16-triazanonadecyl)cyclohexane-1-carboxamido)-3-(naphthalen-2-yl)propanamido)-1-carboxypentyl)carbamoyl)-L-glutamic acid (NOTA-PSMA-CM). The details of the synthesis refer to previous reports [27].^64^CuCl_2_ was obtained as solution in 0.1 M HCl. 50 μL ^64^CuCl_2_ solution, 200 μL NaAc buffer (0.1 M, pH = 5.5) and 4 μL PSMA-CM (4 mM in water, 16 nM) were mixed and the mixture was heated at 95 °C for 15 min. After reaction, the mixture was loaded into the syringe with 2 mL of H_2_O, then passed through a Sep-Pak C18-Light Cartridge (pretreated with 5 mL ethanol and 10 mL H_2_O, respectively) and the C18-Light Cartridge was washed with 5 mL of H_2_O. The product was obtained by eluting the C18-Light Cartridge with 0.5 mL 80% ethanol, diluting with 4 mL 0.9% saline. 

Quality control of ^64^Cu-PSMA-CM was performed by radio-high-performance liquid chromatography (radio-HPLC) and radio-thin-layer-chromatography (radio-TLC). Radio-HPLC was performed on a C18 cartridge (ZORBAX 300SB-C18, 4.6 mm × 250 mm, 5 μm; Agilent, Palo Alto, California, CA, USA) with the mobile phases of (A) H_2_O and (B) acetonitrile mixed with 0.1% trifluoracetic acid (TFA) using a linear AB gradient (15–60% of B in 15 min) with a flow of 1 mL/min and the UV of 280 nm. Radio-TLC was conducted on an AR 2000 systemwith the carrier of filter paper and the eluant of a mixture of saturated EDTA and saline.

### 4.3. In Vitro Stability

^64^Cu-PSMA-CM was incubated in saline or 5% human serum albumin (HSA) at 37 °C and the radiochemical purity was determined by radio-HPLC at different points in time (1 h, 4 h, 12 h, 24 h, and 48 h). For the stability in 5% HSA, 15 μL of mixture was taken out and precipitated by ethanol, filtered, and the supernatant was analyzed.

### 4.4. Partition Coefficient

Amounts of 10 μL of ^64^Cu-PSMA-CM in saline (0.37 MBq), 990 μL of phosphate-buffered saline (PBS, 0.1 M, pH 7.4), and 1mL of octanol were mixed in a tube. The mixture was vortexed for 3 min and centrifuged (3000 rpm × 5 min). Then, five samples (100 μL) from each phase were removed and the radioactivity was measured. The experiment was repeated three times. The partition coefficient value was calculated as below: P = (average of counts in octanol/average of counts in PBS), the value was expressed as log P ± SD.

### 4.5. Cell Culture and Animal Models

Human prostate cancer cell lines (22Rv1 and PC-3) were obtained from the Stem Cell Bank, Chinese Academy of Science. Both cell lines were cultured in RPMI 1640 medium containing 10% fetal bovine serum (FBS) and 1% penicillin–streptomycin (Biological Industries, Shanghai, China). The cells were cultured in a humidified incubator at 37 °C with 5% CO_2_.

Healthy male KM mice and BALB/c nude mice aged 6–8 weeks were acquired from Vital River Laboratory Animal Technology Co., Ltd. (Beijing, China). Healthy male KM mice were used for the pharmacokinetics experiment. About 2 × 10^6^ cells of 22Rv1 in RPMI 1640 medium were subcutaneously injected into the front left of the BALB/c nude mice. When the tumors reached a size of approximately 0.5–1 cm^3^ in volume, the mice were used for further animal studies including biodistribution and micro-PET imaging. All animal experiments were conducted with the approval of the Ethics Committee of Peking University Cancer Hospital.

### 4.6. Western Blotting and Cell Uptake

The Western blotting assay was conducted on 22Rv1 and PC3 cell lines to verify the expression of PSMA. The details are described in the supporting information.

The 22Rv1 and PC-3 cell lines were plated on 24-well plates (2 × 10^5^ cells/well). After incubating for 24 h, the medium was removed and washed with PBS (phosphate buffer saline, 0.01 M). Then, 500 μL medium with 74 KBq of ^64^Cu-PSMA-CM was added to the well plates and co-incubated with cells at 37 °C for 10 min, 30 min, 60 min, and 120 min. The medium was then removed and the cells were washed twice with 1 mL of cold PBS. After that, cells were lysed with 1M NaOH, and then the NaOH solution was collected for analysis. For the blocking experiment, cells were co-incubated with ZJ-43 (1 µg/well), a PSMA inhibitor, for 120 min. Five samples of ^64^Cu-PSMA-CM were taken out and were thought to be standard. The result was expressed as %IA/10^6^ cells. For comparison, the cell uptake experiment of ^64^Cu-PSMA-BCH in 22Rv1 cells was performed.

### 4.7. Binding Affinity Assay

In order to test the HSA protein binding ratio, 10 μL of ^64^Cu-PSMA-CM (0.37 MBq) was added to 0.2 mL of 20% HSA (n = 5) and incubated at 37 °C for 4 h. Then the protein was precipitated by 0.5 mL of ethanol, centrifuged (5000 rpm), washed by saline (2 × 0.2 mL), and centrifuged twice, then measured for the radioactivity of the precipitation and supernatant by a γ-counter. The result was presented as the percentage of precipitation counts/(precipitation counts + supernatant counts). The human serum albumin binding ability of ^64^Cu-PSMA-BCH was measured with the same protocol.

The dissociation constant of ^64^Cu-PSMA-CM was performed on 22Rv1 cells by adding different concentrations of ^64^Cu-PSMA-CM (0.185–74 kBq/mL, 400 μL/well, 4 well/group) to the 24-well plate (2 × 10^5^ cells/well). After incubation for 2 h at 37 °C, the medium was removed, the cells were washed twice with cold PBS and then lysed by 0.5 mL NaOH. The radioactivity of NaOH solution was measured and the dissociation constant was calculated using the one-site total model in Graph Pad software (Graph Pad prism 5). Similarly, the dissociation constant of ^64^Cu-PSMA-CM was determined with the same protocol.

### 4.8. Pharmacokinetics in Blood

An amount of 3.7 MBq (250 μL) of ^64^Cu-PSMA-CM was intravenously injected into Babl/c male mice (n = 4). Blood was collected from the ophthalmic artery at different time points, then the radioactivity of blood was weighed and measured by a γ-counter. An injection of 2.5 μL was taken out as standard. The results were expressed as the percentage of injected dose per gram (%ID/g). 

A two-phase decay Equation (1) in Graph Pad software (Graph Pad prism 5) was used to serve as the two-compartment model which models biological process of the probe by fitting the ID%/g versus to time of ^64^Cu-PMA-CM to describe blood pharmacokinetics.

C_t_ = Ae^−*α*t^ + Be^−*β*t^ (1)

where A and B are relevant constants, and *α* and *β* are rate constants which were used to calculate as ln(2)/*α* and ln(2)/*β* for the half-life of distribution phase and elimination phase, respectively.

### 4.9. Biodistribution

Twelve 22Rv1 tumor model mice were randomly divided into four groups and then ^64^Cu-PSMA-CM (200 μL, 0.74 MBq/mouse) was injected via tail vein. After 1 h, 6 h, 24 h, and 48 h, the mice were sacrificed by cervical dislocation. Organs of interest including blood, heart, liver, spleen, lung, kidney, small intestine, large intestine, muscle, brain, and 22Rv1 tumor were collected. The organs were then weighed and measured for their radioactivity. Five samples of the 1% injection volume were taken out as a standard. The final uptake values of each organ were expressed as the percentage of injected dose per gram (%ID/g).

### 4.10. Micro-PET Imaging and Immunohistochemical Staining

An amount of 200 μL of ^64^Cu-PSMA-CM (9.32 MBq) was injected into mice bearing 22Rv1 xenograft tumors via tail vein. For blocking, the mice were co-injected with 50 μg of ZJ-43. Imaging was performed on Super Nova PET/CT (PINGSENG, Shanghai, China). The mice were anaesthetized with 3% (*v*/*v*) isoflurane, then fixed on the bed at 4 h, 8 h, 12 h, 24 h, and 48 h p.i. Micro-PET scans were conducted with continuous 1% (*v*/*v*) isoflurane for 15 min. The images of ^64^Cu-PSMA-CM were obtained at different time points and SUV mean values of regions of interest (ROIs) over the heart, kidney, muscle, liver, and tumor were collected. The ratio of tumor-to-non-target organs was then calculated at different time points. To better assess its anti-tumor efficiency in vivo, a comparison with ^64^Cu-PSMA-BCH (5.55 MBq/mouse) of micro-PET imaging was performed in mice bearing 22Rv1 tumor models at 4 h p.i., 12 h p.i., and 24 h p.i. The mean standardized uptake value (SUVmean) in each organ was calculated according to following Formula (2) [7].

SUV = Activity VOI (MBq/mL)/Injected dose (MBq) × Body weight (g)(2)

After micro-PET imaging, the 22Rv1 mice were sacrificed by cervical dislocation, and the 22Rv1 tumor tissues were quickly collected and immersed in formalin solution for further immunohistochemical staining. The details are presented in the supporting information.

### 4.11. Estimation of Radiation Dosimetry in Human Organs

The fraction of radioactivity uptake in human tissues was calculated according to the biodistribution results in mice bearing 22Rv1 tumors. The time–activity curves of various organs and the whole body were obtained and then calculated the area under the curves (AUCs) of different organs with origin 2019b software (OriginLab, Northampton, MA, USA). The results were further analyzed with OLINDA/EXM software (version 2.2; HERMES Medical Solutions AB) to estimate the radiation dosimetry of each organ and effective dose.

### 4.12. Statistical Analysis

All quantified data were expressed as the mean ± SD. The significant differences of cell uptake and human serum albumin binding affinity were calculated by two-tailed, paired t-test. Quantified ROI data based on the results of micro-PET imaging were analyzed using two-way ANOVA. Statistical analysis was performed using the Microsoft Excel 2016 software program (Microsoft Corporation) or Graph Pad software (Graph Pad prism 5). The *p* values less than 0.05 were considered statistically significant.

## 5. Conclusions

In this study, we successfully developed an albumin-based PSMA probe conjugated with MPA, ^64^Cu-PSMA-CM. It exhibited good physicochemical and biological properties. It showed a longer half-life in blood, a high affinity to PSMA, and a higher uptake in PSMA (+) tumors—which effectively monitored the change of radioactivity accumulation of PSMA-targeted tracers in tumors. The probe offers a good strategy and was initially verified for the potential to develop radiotracers for PRLT for prostate cancer. 

## Figures and Tables

**Table 1:** Human organ radiation dosimetry estimation of ^64^Cu-PSMA-CM.

Target Organ	Absorbed Dose (mGy/MBq)
Adrenals	3.94 × 10^−2^
Brain	2.84 × 10^−2^
Esophagus	2.95 × 10^−2^
Eyes	2.53 × 10^−2^
Gallbladder Wall	4.14 × 10^−2^
Left colon	3.16 × 10^−2^
Small Intestine	4.00 × 10^−2^
Stomach Wall	3.09 × 10^−2^
Right colon	3.33 × 10^−2^
Rectum	3.80 × 10^−2^
Heart Wall	3.25 × 10^−2^
Kidneys	9.97 × 10^−2^
Liver	1.07 × 10^−1^
Lungs	3.27 × 10^−2^
Pancreas	3.32 × 10^−2^
Prostate	3.08 × 10^−2^
Salivary Glands	2.80 × 10^−2^
Red Marrow	2.40 × 10^−2^
Osteogenic Cells	3.46 × 10^−2^
Spleen	1.74 × 10^−2^
Testes	2.73 × 10^−2^
Thymus	2.83 × 10^−2^
Thyroid	2.82 × 10^−2^
Urinary Bladder Wall	3.06 × 10^−2^
Total Body	3.09 × 10^−2^
Effective Dose (mSv/MBq)	2.76 × 10^−2^

## Data Availability

Data is contained within the article and supplementary material.

## Supplementary Materials

The following supporting information can be downloaded at: https://www.mdpi.com/article/10.3390/ph15050513/s1, Figure S1: Quality control of ^64^Cu-PSMA-CM. (a) The radio-chemical purification of ^64^Cu-PSMA-CM by Radio-HPLC analysis before purification. The radio-chemical purification of ^64^Cu-PSMA-CM after purification by Radio-HPLC (b) and radio-TLC (c) analysis; Figure S2: The SUV mean values of ^64^Cu-PSMA-CM (a) and ^64^Cu-PSMA-BCH (b) in organs and 22Rv1 tumor at different time points according to images in 22Rv1 xenograft nude mice. ***: *p* < 0.001; Figure S3: The tumor-to-kidney (a) and tumor-to-liver (b) ratios of ^64^Cu-PSMA-CM and ^64^Cu-PSMA-BCH at different time points according to images in 22Rv1 xenograft nude mice; Table S1: Biodistribution of ^64^Cu-PSMA-CM in 22Rv1 male mice (ID%/g, X ± SD, n = 3).
