# Supplementary material for: An Albumin-Binding PSMA Ligand with Higher Tumor Accumulation for PET Imaging of Prostate Cancer
Source: Pharmaceuticals (Basel). 2022 Apr 22;15(5):513. doi: 10.3390/ph15050513 (PMC9143078; doi:10.3390/ph15050513)
Supplement: Supplementary file 1 [file pharmaceuticals-15-00513-s001.zip › pharmaceuticals-1638784-supplementary.pdf]

An albumin-binding PSMA ligand with higher tumor accumulation for PET imaging of prostate cancer

Ya'nan Ren [1,2,†], Teli Liu [1,†], Chen Liu [1], Xiaoyi Guo [1], Feng Wang [1], Hua Zhu [1,2] and Zhi Yang [1,2,*]

[1] Key Laboratory of Carcinogenesis and Translational Research (Ministry of Education/Beijing), Key Laboratory for Research and Evaluation of Radiopharmaceuticals (National Medical Products Administration), Department of Nuclear Medicine, Peking University Cancer Hospital & Institute, Beijing 100142, China; yananren123@126.com (Y.R.); liuteli123321@163.com (T.L.); chanmx@hotmail.com (C.L.); 1911110575@bjmu.edu.cn (X.G.); windtigerwf@163.com (F.W.)

[2] School of Medicine, Guizhou University, Guiyang 550025, China

* Correspondence: zhuhuananjing@163.com (H.Z.); pekyz@163.com (Z.Y.)

† These authors contributed equally to this work.

Supporting information:

Materials and Methods

Western blotting

For western blot analysis, 22Rv1 and PC3 cells in each dish were lysed with 500 μL RIPA lysate buffer (40 mM Tris-HCl (pH 7.4) containing 150 mM NaCl and 1% (v/v) Triton X-100, supplemented with a cocktail of protease inhibitors and PMSF) for 5 min. The cell lysate was centrifuged for 5 min at 14000 g at 4 ℃ and the supernatant cellular proteins were collected. Equal amounts of supernatant were separated to extract protein in 10% SDS-PAGE gels, then transferred to a polyvinylidene fluoride (PVDF) membranes. After blocking with 5% non-fat milk dissolved in TBST solution, the membranes were incubated with primary antibody rabbit monoclonal anti-PSMA antibody (ab133579, Abcam, China) at 4°C overnight. Removing primary antibody, the membranes were washed three times with TBST, then co- incubated with HRP-conjugated goat anti-rabbit IgG secondary antibody (AS014, ABclonal, China) for 1 h at room temperature. After washing with TBST, the blots were developed using the Super Enhanced chemiluminescence detection kit (Applygen Technologies Inc., Beijing, China). Protein bands were visualized after exposure of the membrane to imaging system (Amersham Imager 680, GE Healthcare, America).

Immunohistochemical Staining

Immunohistochemical studies were performed on formalin-fixed, paraffin-embedded (FFPE) 4um specimens using standard Envision techniques, with tissue sections dewaxed in xylene and rehydrated in graded alcohol. Endogenous peroxidase was blocked with 3% dioxygenated water. Sections were subjected to antigen repair using microwaves. After blocking in goat serum for 1 hour, sections were incubated overnight with primary antibody PSMA (ab133579, Abcam, China) and primary antibodies were prepared at a dilution of 1:50000 in a humidified chamber at 4°C. The next day, after 30 minutes of rewarming at room temperature, the sections were stained using the Super sensitive polymer HRP IHC detection system (ZSGB-BIO PV-6000) according to the manufacturer's instructions. The entire specimen was examined by light microscopy and scanned for images using an Aperio Versa 200, Leica.

Results

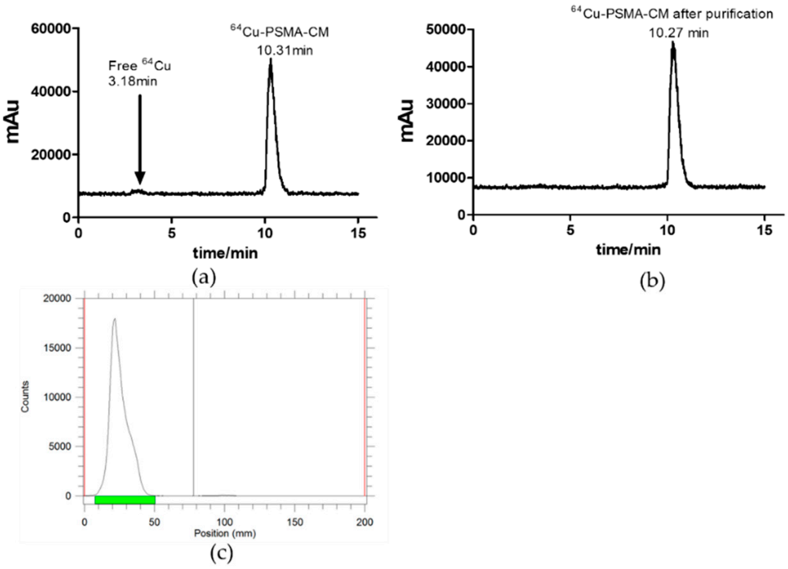

Figure S1. Quality control of ^{64}Cu-PSMA-CM. **(a)** The radio-chemical purification of ^{64}Cu-PSMA-CM by Radio-HPLC analysis before purification. The radio-chemical purification of ^{64}Cu-PSMA-CM after purification by Radio-HPLC **(b)** and radio-TLC **(c)** analysis.

Table S1. Biodistribution of ^{64}Cu-PSMA-CM in 22Rv1 male mice (ID%/g, X±SD, n=3)

	1 h	6 h	12 h	24 h
Heart	4.49±0.47	4.16±0.62	1.96±0.35	0.95±0.21
Liver	5.34±1.13	5.24±0.59	8.50±1.06	6.14±0.23
Lung	9.91±0.43	8.47±1.45	6.28±0.72	5.60±0.23
Kidney	95.84±3.33	26.78±0.69	14.19±1.40	8.92±0.41
Spleen	8.48±3.35	4.63±1.13	2.65±0.55	2.31±0.26
Muscle	1.15±0.09	1.22±0.25	1.24±0.77	-
LI	3.75±0.11	2.85±0.06	4.32±0.69	2.81±0.35
SI	6.00±0.35	5.71±0.65	3.45±0.70	2.81±0.93
tumor	8.09±1.66	8.51±1.99	14.29±1.44	10.57±1.23
Blood	16.95±3.05	12.33±2.83	5.41±0.94	2.83±0.41
Brain	0.69±0.07	0.47±0.05	0.44±0.06	-

LI: large intestine; SI: small intestine.

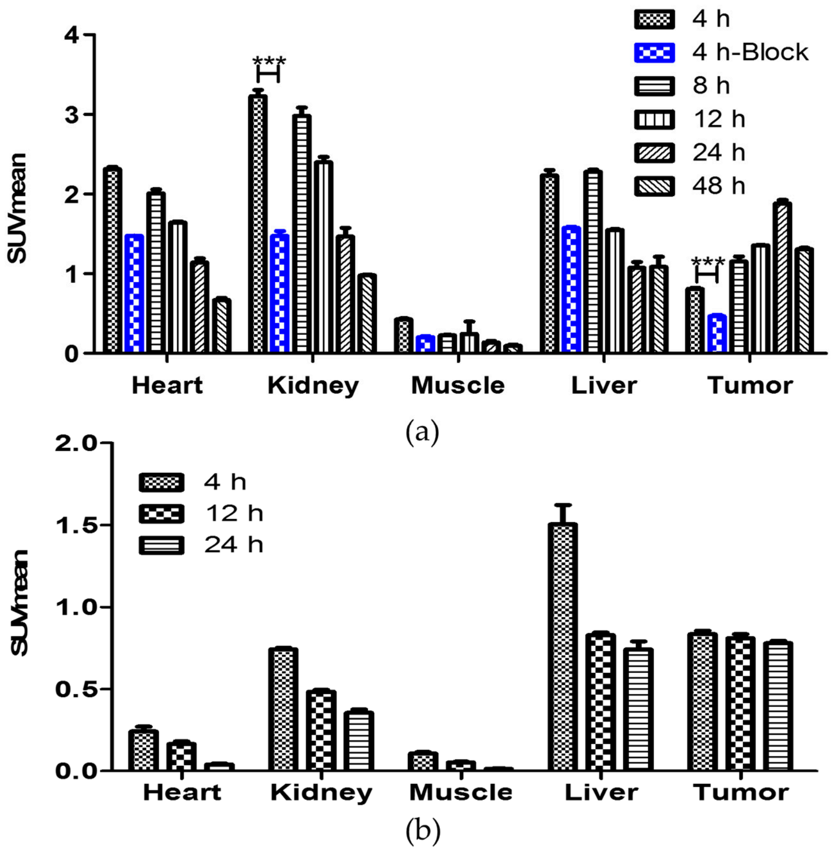

Figure S2. The SUVmean values of ^{64}Cu-PSMA-CM **(a)** and ^{64}Cu-PSMA-BCH **(b)** in organs and 22Rv1 tumor at different time points according to images in 22Rv1 xenograft nude mice. ***: $P < 0.001$

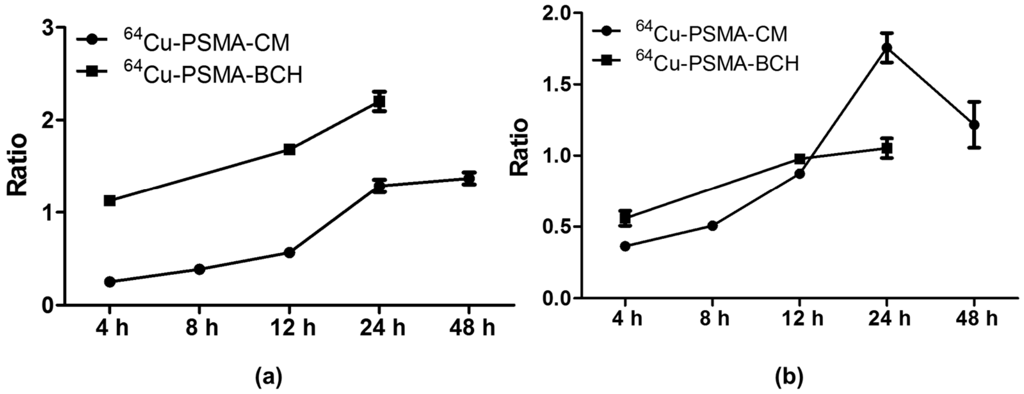

Figure S3. The tumor-to-kidney **(a)** and tumor-to-liver **(b)** ratios of ^{64}Cu-PSMA-CM and ^{64}Cu-PSMA-BCH at different time points according to images in 22Rv1 xenograft nude mice.
